# Management of Arrhythmias in Heart Failure

**DOI:** 10.3390/jcdd4010003

**Published:** 2017-02-28

**Authors:** Daniele Masarone, Giuseppe Limongelli, Marta Rubino, Fabio Valente, Rossella Vastarella, Ernesto Ammendola, Rita Gravino, Marina Verrengia, Gemma Salerno, Giuseppe Pacileo

**Affiliations:** Cardiologia SUN—Heart Failure Unit, Department of Cardiothoracic Sciences, Second University of Naples, via L. Bianchi, Naples 80100, Italy; limongelligiusepppe@libero.it (G.L.); rubinomarta@libero.it (M.R.); fabio.valente@alice.it (F.V.); rossellavastarella86@gmail.com (R.V.); ammendolaernesto@libero.it (E.A.); ritagravino@libero.it (R.G.); mariverr@yahoo.it (M.V.); gemmsalerno@hotmail.com (G.S.); gpacile@tin.it (G.P.)

**Keywords:** heart failure, tachyarrhythmias, bradyarrhythmias, sudden cardiac death

## Abstract

Heart failure patients are predisposed to develop arrhythmias. Supraventricular arrhythmias can exacerbate the heart failure symptoms by decreasing the effective cardiac output and their control require pharmacological, electrical, or catheter-based intervention. In the setting of atrial flutter or atrial fibrillation, anticoagulation becomes paramount to prevent systemic or cerebral embolism. Patients with heart failure are also prone to develop ventricular arrhythmias that can present a challenge to the managing clinician. The management strategy depends on the type of arrhythmia, the underlying structural heart disease, the severity of heart failure, and the range from optimization of heart failure therapy to catheter ablation. Patients with heart failure, irrespective of ejection fraction are at high risk for developing sudden cardiac death, however risk stratification is a clinical challenge and requires a multiparametric evaluation for identification of patients who should undergo implantation of a cardioverter defibrillator. Finally, patients with heart failure can also develop symptomatic bradycardia, caused by sinus node dysfunction or atrio-ventricular block. The treatment of bradycardia in these patients with pacing is usually straightforward but needs some specific issue.

## 1. Introduction

Arrhythmias confers a substantial risk of mortality and morbidity in patients with heart failure (HF), and this represents a major health care burden worldwide. There are at least 15 million patients with HF in Europe [1] and 6 million patients in USA [2], with an overall prevalence ranging between 2% and 3% that increases sharply after 75 years of age, reaching 10%–20% among those in the eighth decade of life. HF hospitalizations are increasing, and many of these may be related to supraventricular arrhythmias (SVAs) [3] such as atrial fibrillation (AF). Atrial fibrillation per se contributes to an increased risk of mortality and morbidity of stroke and thromboembolism, and silent AF is common among patients with HF [4], not infrequently leading to a first presentation of AF with an ischemic stroke [5]. Sudden cardiac death (SCD) is also a major cause of mortality among HF patients and is commonly related to cardiac arrhythmias [6], particularly ventricular arrhythmias (VAs). Finally, bradyarrhytmias are common in HF and may include sinus node dysfunction, tachy-brady syndrome, and conduction disease [7]. This review focuses on the management of arrhythmias, both tachyarrhythmias and bradyarrhytmias, in patients with HF.

## 2. Pathophysiology of Tachyarrhytmias in HF

Highly complex, interactive, and dynamic changes in mechanical, structural, neurohumoral, metabolic, and electrophysiological properties collectively predispose the failing heart to tachyarrhytmias (Table 1), which increase in complexity with the severity of left ventricular dysfunction [8].

### 2.1. Structural and Hemodynamic Abnormalities

#### 2.1.1. Myocardial

Possibly the best understood relationship between arrhythmias and structural changes is represented by post-myocardial infarction cardiomyopathy and reentry.

In these patients there are focal areas of post-infarct scar with creating a heterogeneous environment that promote the development of ventricular arrhythmias due to anisotropy [9,10] and reentry [11].

#### 2.1.2. Chamber Hypertrophy and Stretch

In patients with HF, numerous compensatory mechanisms are active to improve cardiac output, but these mechanisms can contribute to arrhythmogenic milieu. Left ventricular hypertrophy is known due to pro arrhythmic electrophysiological changes (reduced cell-cell coupling, reduction of membrane potentials, sub-endocardial ischemia) [12]. Other factors favoring arrhythmias include increases preload and afterload, which reduce the repolarization phase of action potential [13]. Increases in preload and afterload, as well as high left ventricular filling pressure can also contribute to the arrhythmogenic substrate determining the onset of subendocardial ischemia [14]. 

### 2.2. Metabolic Abnormalities

#### 2.2.1. Neurohormonal Activation

The activation of the neurohormonal systems is responsible for a variety of proarrhythmic changes. The increased plasma levels of adrenaline and noradrenaline plays an important role in the pathophysiology of HF and are believed to be partly responsible for the beneficial effects of beta-blockers and angiotensin converting enzyme (ACE) on the death sudden [15,16]. Meredith and colleagues [17] have shown a significant increase of norepinephrine relapse in patients with HF that may contribute to arrhythmias. Moreover, Cao and colleagues describe an association between the density of nerve sympathetic fibers (which are increased in patients with HF) and the risk of Vas [18].

#### 2.2.2. Electrolyte Abnormalities

Hyperkalemia is common in patients with HF and is often linked to the use of ACE inhibitors, angiotensin receptor blockers and aldosterone antagonists. Hyperkalemia leading to slow the ascent phase of action potential that can cause atrioventricular block [19]. Although hypokalemia is very common in HF patients and may be secondary to increased activity of the renin-angiotensin system or the use of loop diuretics [20]. The result is a more automaticity in Purkinje fibers and a rapid increase repolarization, with consequent onset of VT/VF [21]. Hypomagnesemia it is also common in patients with HF, however, the association between hypomagnesemia and VAs is not as strong as for potassium abnormalities [22].

### 2.3. Pharmacologic Agents

The drugs that interfere with neuro-hormonal system can often contribute to the pro-arrhythmic milieu. As mentioned diuretic therapy, ACE inhibitors and aldosterone antagonists often cause electrolyte imbalances that contribute significantly to arrhythmogenesis and can increase the proarrhythmic effect of other drugs [23].

Inotropic agents that may be required in patients with acute HF and hypoperfusion especially in the presence of electrolyte abnormalities may further increase the risk of arrhythmias.

### 2.4. Electrophysiologic Changes in Heart Failure

#### 2.4.1. Action Potential Prolongation

An electrophysiological hallmark of cells and tissues isolated from hypertrophied and failing hearts is a prolonged action potential duration, reflecting delayed terminal repolarization of the cardiac myocyte [24]. These cellular electrophysiological changes were mechanistically linked to downregulation of repolarizing potassium currents, an increase in late sodium current density, as well as major changes in intracellular calcium.

Studies in humans and animal models of HF showed delayed global repolarization and enhanced temporal repolarization dynamics using clinical noninvasive metrics, such as the QT-interval variability index and T-wave alternans on the surface electrocardiogram [25]. However, despite these studies, a direct mechanistic link between repolarization changes observed in isolated myocytes or on the body surface and arrhythmia genesis remains lacking.

#### 2.4.2. Calcium Handling

Among the numerous electrophysiological changes that are present in the clinical syndrome of HF, none is so closely involved in arrhythmogenesis as calcium homeostasis. The calcium channel L-type voltage-dependent are the main source of calcium influx into myocardial cells and are responsible for its release from the sarcoplasmic reticulum. These channels have a decreased function in patients with HF, resulting in a prolongation of the action potential [26,27]. Recent studies have demonstrated a downregulation of SERCA2a calcium reuptake proteins, as well as an increase in the ryanodine receptor during diastole, resulting in calcium “leak” [28].

Slow calcium leak during diastole increased calcium release during the sarcoplasmic reticulum stimulation, and decreased uptake by SERCA2a finally establishes an intracellular calcium overload that favors the arrhythmogenesis [29]. 

In addition blocking transient inward current (Iti) with tetracaine, Verker et al. showed a complete suppression of delayed after depolarization confirming the role of these currents in the arrhythmogenic process [30]. Finally, the increased activity of the beta-adrenergic receptor may contribute to spontaneous release of calcium from the sarcoplasmic reticulum [31].

#### 2.4.3. Role of the Sodium–Calcium Exchanger

The sarcolemmal sodium and calcium exchanger normally regulates the flow of calcium in cardiomyocyte facilitating the relaxation [32].

In experimental models of HF it was reported an increase of the sodium and calcium exchanger with a possible role in the genesis of arrhythmias [33].

#### 2.4.4. Voltage-Dependent Potassium Channels

In patients with advanced HF there is a reduced function of both the external potassium current (Ito) and inward rectifier potassium current (IK1), these channels play a key role in the genesis of the action potential [34]. Beuckelmann and colleagues [35] were the first to identify a decrease in potassium currents in HF patients although their role in arrhythmogenesis remained controversial.

## 3. Pathophysiology of Bradyarrhytmias in HF

Bradyarrhythmias may be due either to a reduction in heart rate or a block in the impulse conduction after its formation.

### 3.1. Sinus Node Dysfunction

Symptomatic sinus bradycardia is due to a reduction in impulse formation in the sinus node or lack of impulse conduction from the sinus node to the atrial tissue. In most cases, the nodal dysfunction is caused by progressive fibrosis of nodal cells due to aging; however, any process that causes damage to the sinoatrial node (e.g., nodal artery atherosclerosis, infiltrative diseases or collagen diseases), may be contribute to nodal dysfunction [36]. The sinoatrial dysfunction can occur with episodes of sinoatrial block in a significant fraction of patients, in addition approximately 50% of patients with sick sinus syndrome develop alternating bradycardia and tachycardia, also known as tachy-brady syndrome [37]. The most common tachyarrhythmias are atrial fibrillation or flutter with rapid ventricular response. These tachyarrhythmias are more common in older patients with advanced sinoatrial nodal disease in whom sinoatrial node fibrosis may favor reentrant beats or tachycardia.

### 3.2. Atrio-Ventricular Node Dysfunction

First-degree atrio-ventricular block is not associated with bradycardia except in the setting of sinus bradycardia. First-degree atrio-ventricular block is commonly associated with damage and/or fibrosis of the atrio-ventricular node, but it can also be due to beta-blockers therapy [38]. Like 1st degree atrio-ventricular block, Type I second-degree atrio-ventricular block can occur in normal individuals and is common during sleep in 4%–6% of cases [39]. It may be particularly prevalent among patients with HF and obstructive sleep apnea, in which therapy with positive airway pressure often alleviates the finding. Type I second-degree atrio-ventricular block can lead to loss of atrio-ventricular synchrony, although this is typically not problematic unless a substantial first degree atrio-ventricular is also present. Type II second-degree atrio-ventricular block is associated with disease of the conduction system below the atrio-ventricular node, and as such has a higher likelihood of progression to complete atrio-ventricular block [40]. In patients with second degree type II atrioventricular block is common a slowing of impulse conduction on His-Purkinje system, this is demonstrated during electrophysiological study by an abnormally long H-V interval [41]. High-grade atrio-ventricular block and complete heart block represent the most severe forms of atrio-ventricular nodal disease and are usually associated with underlying heart disease [42].

## 4. Clinical Impact of Tachyarrhythmias in HF

**Figure 1:**
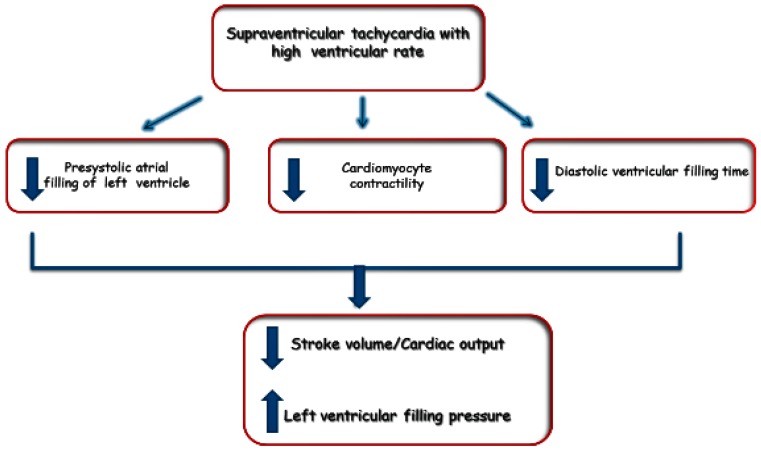
Hemodynamic effects of supraventricular tachycardia.

Although any SVAs may occur in patients with HF, the most common is AF—indeed, AF and congestive HF commonly occur together and each may exacerbate the other [43]. The Framingham Heart Study demonstrated that each condition increases the risk for developing the other. There is a 15% 5-year incidence of new-onset HF among patients with AF; the converse is also true, with a 25% 5-year incidence of new AF in patients with HF [44]. The prevalence of AF progresses with worsening HF with a prevalence of 10% in HF patients in the New York Heart Association (NYHA) Classes I and II, and up to 50% in NYHA Class IV. HF and AF share the presence of common risk factors, such as age, high blood pressure, diabetes mellitus, obesity and valvular dysfunction [45]. However, experimental and clinical data document a substrate of the atrial myocardium for the AF in patients with HF regardless of the shared risk factors. HF patients may also experience other type of SVAs (e.g., focal atrial tachycardias and atrial macroreentrant arrhythmias), sometimes particularly challenging to differentiate from HF-related sinus tachycardia. SVAs can cause tachycardia-related cardiomyopathy, as can AF and atrial flutter (AFL), and ventricular rate control is paramount to treatment of the resulting cardiomyopathy [46]. Likewise, in patients with HF-reduced ejection fraction (HFrEF), SVAs can have detrimental effects on cardiac hemodynamics with exacerbation of HF syndrome (Figure 1); on the other hand, mostly patients with HF-preserved ejection fraction (HFpEF) are dependent on adequate ventricular filling. For this reason, onset of AF (in which the normal atrial contraction is replaced by ineffective quivering of the atria) can cause a rapid onset of HF symptoms [47].

In addition, atrial tachyarrhythmias may influence cardiac resynchronization therapy response (i.e., reduction of pacing rates) and sometimes induce inappropriate therapy from the implantable cardioverter defibrillator (ICD). 

Finally, patients with AF and AFL bears a increased risk of ischemic stroke and systemic embolism compared to sinus rhythm, and this risk is further increased in patients with HF [48].

More than 80% of patients with HFrEF have frequent and complex VAs, as documented on Holter monitoring, and almost 50% demonstrate runs of non-sustained ventricular tachycardia (non-sustained VT).

The presence and severity of VAs increase along with the severity of HF, but their value to predict SCD is unclear [49].

Isolated premature ventricular complexes (PVCs) may be asymptomatic, whereas more complex VAs may be accompanied by symptoms as palpitations, lightheadedness, presyncope or syncope, and a worsening of HF [50].

Of concern is the association of NSVT with sustained ventricular tachycardia (sustained VT) and ventricular fibrillation (VF), the usual mechanisms for SCD [51].

However, the role of VAs to predict sudden death is unclear; for this reason, SCD risk stratification in HF patients requires a multiparametric evaluation (see below).

## 5. Clinical Impact of Bradyarrhythmias in HF

Arrhythmias in the HF population are not confined to tachyarrhythmias. While the ever-increasing percentage of HF patients implanted with devices capable of pacing precludes quantification of bradycardic risk at the current time, earlier registries illustrated that bradyarrhythmias with electromechanical dissociation were the mechanism of SCD in a significant number of selected HF patients [52]. Additionally, reversible sinus node dysfunction and atrio-ventricular block may result from drug toxicity [53] (e.g., digoxin, beta-blockers) or electrolyte disorders and can induce worsening of HF symptoms.

## 6. Management of Supraventricular Tachyarrhythmias in HF

### 6.1. Management of Atrial Fibrillation

According to guidelines of European Society of Cardiology (ESC) [54], AF is classified clinically as paroxysmal (self-terminating episodes lasting <7 days), persistent (non-self-terminating episodes lasting 7 days to 6 months), long-standing persistent (lasting longer than 1 year), and permanent (AF for which attempts at termination have failed or which is an accepted rhythm).

Paroxysms of AF or runs of rapid ventricular response may instigate HF exacerbation or be caused by HF exacerbation; for this reason, treatment goals of HF must address AF. Fortunately, many of the same medications used to treat HF also treat AF: β-blockers and digoxin will help control heart rate [55], while ACE inhibitors may lead to reverse remodeling and reductions in AF burden [56]. Antiarrhythmic drugs, on the other hand, do not have a primary role in the treatment of HF; hence, the rhythm control strategy, although effective in treating AF, has shown no survival benefit in HF patients. AF treatment strategies can be divided into rate control and rhythm control; in addition, risk stratification for stroke is central in the management of HF patients with AF [57].

#### 6.1.1. Rate Control

Rate control in the acute HF can be achieved with intravenous calcium channel blockers (e.g., verapamil 0.075/0.15 mg/kg iv in 2 min, then 0.005 mg/kg/min or switch to 280–480 mg/day orally) or β-blockers (e.g., metoprolol 2.5–5 mg iv in 2 min every 5 min, maximum 15 mg, then 50–200 mg/day orally). These medications should be used with caution in the hypotensive or low normotensive patient, since hypotension may be exacerbated by negative inotropic medications [58]. Infusions of short-acting medications such as esmolol (500 mcg/kg iv in 1 min, then 50–300 mcg/kg/min) may be useful to allow for a quickly reversible therapeutic trial, although such an approach is not evidence-based. The strategy for long-term rate control has more evidence basis than acute treatment. β-Blockers serve a dual role in treating HF and controlling rate, whereas digoxin is beneficial as an adjunct and reduces HF exacerbations [59].

The target for heart rate in the AF Follow-up Investigation of Rhythm Management (AFFIRM) trial was defined as less than 80 beats per minute at rest, and 110 beats per minute during a 6 min walk test; however, the Rate Control Efficacy (RACE-2) trial randomized patients to conventional strict control (<80 beats per minute at rest) or to a “lenient” approach (<110 beats per minute at rest) and found there to be no significant difference in mortality, NYHA class, and HF hospitalizations [60].

#### 6.1.2. Rhythm Control

Maintenance of sinus rhythm with a strategy that may include a combination of rate control therapy, antiarrhythmic medication, cardioversion, and radiofrequency ablation can be especially challenging in the HF patient. Several factors make rhythm control more difficult to achieve in this patient population, including left atrium enlargement, comorbidities (e.g., hypertension, obstructive sleep apnea), and increased sympathetic tone [61]. Nevertheless, despite lack of advantages over rate control in mortality, stroke reduction, or hospitalization, rhythm control has a vital role in treating symptomatic patients.

Acute rhythm control is indicated in hemodynamically unstable patients and can be achieved with either pharmacotherapy or direct current cardioversion. Electrical cardioversion with 200 joules biphasic energy is over 94% effective, and there is little benefit and potential harm (from repeat cardioversion) in using lower energy levels [62]. In HF patients, care must be taken to assess for hypokalemia and/or digoxin toxicity prior to cardioversion, in patients with hypokalemia potassium repletion is indicated to reduce the risk of VAs.

Pharmacological cardioversion when done safely provides a less invasive option, but a lower success rate with higher risk of adverse drug effects.

Ibutilide (0.01 mg/kg iv with a second dose 10 min later if necessary) is approximately 50% effective at cardioversion; however, the risk of torsades de pointes increases to 11.4% compared to 3.6% in non-HF patients [63]. Class Ic agents flecainide and propafenone are contraindicated in the structural heart disease because of pro-arrhythmia and should not be used for chemical cardioversion. Amiodarone (150 mg in 10 min, then 1 mg/min for 6 h, then 0.5 mg/min for 18 h) remains an option for medical cardioversion with restoration of sinus rhythm in 80% of patients [64]. The only other antiarrhythmic medication that has demonstrated safety in HF in large randomized trials is dofetilide (125–500 mcg once every 12 h); however, because of a 3% risk of torsades, inpatient hospitalization to monitor for QT prolongation during initiation is mandatory [65].

#### 6.1.3. Rate Control versus Rhythm Control

The landmark study comparing rate control to rhythm control is the AFFIRM trial, reported in 2002. In this study, there was no difference in mortality or thromboembolic events between the two groups with 5 years of follow-up [66]. More recently, the Atrial Fibrillation and Congestive Heart Failure Trial (AF-CHF) compared rhythm control versus rate control in 1376 patients with EF less than 35% and clinical symptom of HF. There was no difference in death, stroke, or worsening heart failure between the two groups at 5 years of follow-up. Freedom from AF was achieved in just 20% in the rate control arm and 70% in the rhythm control arm [67].

While rhythm control does not lead to decreased mortality or HF, it remains a preferred option in symptomatic patients. In conclusion, the choice between a strategy of rhythm versus rate control should be guided by management of symptoms and patient preference, with no significant difference in mortality or stroke demonstrated in clinical trials.

#### 6.1.4. Anticoagulation

Reduction in risk for stroke in HF patients with AF requires systemic anticoagulation in most patients with structural heart disease. HF itself carries a 2.8% annual risk of stroke in the CHADS-2 validation study, and common comorbidities such as hypertension and diabetes can increase the risk to 5.8% per year [68].

The more recent CHA^2^DS^2^-VASc score, developed in a Danish registry [69], offers a more refined risk stratification, by adding an extra point for female gender and vascular disease, and a two-tier point system for age: 1 point for age 65–75 years and 2 for age over 75 years.

This results in reclassification of the lower-risk patients in the CHADS-2 system, with 39% of those deemed low risk by the CHADS-2 database reclassified as intermediate risk by CHA2DS2-VASc, and 21% reclassified as high risk, with all these patients warranting anticoagulation.

The American College of Cardiology/American Heart Association/Heart Rhythm Society guidelines recommend the CHA2DS2-VASc score as first line in risk stratification for stroke and indicate to start anticoagulation in patients with intermediate or high risk for stroke [70].

The drugs of choice are novel oral anticoagulant while the use of vitamin K antagonists is indicated in patients with mechanical valve, left ventricular thrombus, and contraindication to novel oral anticoagulant [71].

#### 6.1.5. Catheter Ablation

AF ablation with pulmonary vein isolation remains a viable treatment option for symptomatic AF that cannot be treated adequately with antiarrhythmic therapy.

HF patients often have multiple comorbidities and left atrial enlargement, making them unfavorable ablation candidates; however, single-center studies have demonstrated similar efficacy in catheter ablation for AF in HF patients as compared with normal controls, with freedom of AF at 1 year around 70%. Hsu et al. found that in the 86% of patients with inadequate rate control, a 20% increase in EF was achieved with AF ablation [72], which raises the possibility that tachycardia-related cardiomyopathy plays a larger role in HF with AF than generally appreciated.

An intriguing study, the Pulmonary Vein Antrum Isolation versus Atrio Ventricular Node Ablation with Biventricular Pacing for Treatment of Atrial Fibrillation in Patients with Congestive Heart Failure (PABA-CHF), demonstrated that pulmonary vein isolation resulted in an improved 6 min walking distance, increased ejection fraction, and improved symptoms compared to atrio-ventricular node ablation and pacing [73]. While pulmonary vein isolation can be valuable in reducing the frequency of AF in paroxysmal AF, the efficacy is much lower in patients with long-term persistent or permanent AF. If rate control cannot be achieved medically, then atrio-ventricular node ablation with biventricular pacing remains a viable option [74].

In our opinion in patients with HFrEF, due the low efficacy rate of AF ablation, the better option was atrio-ventricular node ablation with biventricular pacing; vice versa, in patients with HFpEF or HF mild reduction ejection fraction (HFmEF), particularly if left atrial volume index is lower than 40 mL/mq, AF ablation represents the best strategy. 

### 6.2. Management of Atrial Flutter

In 1999, Alboni et al. took hemodynamic measurements of 23 consecutive patients in AFL and sinus rhythm and demonstrated increased atrial pressures with a subsequent fall in systemic systolic pressure and rise in diastolic pressures [75]. Consequently, like AF, AFL may lead to HF exacerbation. Treatment strategies for rate control and anticoagulation for AFL are often grouped under the same approaches as for AF and are similar to those discussed in the previous section. The most fundamental difference in AFL as compared to AF is that it is a more organized rhythm and more difficult to rate control adequately [76], and because of this, rhythm control is often the preferred strategy. Fortunately, in contrast to AF, catheter-based treatment for typical AFL flutter is highly effective [77], and often curative, and the guidelines reflect this by making initial catheter ablation for recurrent symptomatic typical atrial flutter a recommended treatment strategy.

### 6.3. Management of Other Type of Supraventricular Arrhytmias

Patients with HF can develop paroxysmal SVAs that are otherwise observed in the healthy population [78]. They may present with atrio-ventricular nodal reentrant tachycardia (AVNRT), atrio-ventricular tachycardia (AVRT) mediated by bypass tracts or atrial tachycardias (AT).

Such arrhythmias are generally treated in a similar fashion as in patients without HF; however, generally, the threshold for performing curative radiofrequency ablation in HF patients should be lower for those arrhythmias that are easily amenable to such therapy (i.e., AVNRT, AVRT).

## 7. Management of Ventricular Tachyarrhythmias in HF

### 7.1. Pharmacologic Management

In most patients with HF PVCs are asymptomatic, in those patients, correction of electrolyte abnormalities (particularly low serum potassium and magnesium), withdrawal of agents that might provoke arrhythmias and optimization of HF pharmacological therapy is indicated; vice versa, treatment of arrhythmias per se is generally not indicated [79].

Non-sustained VT will be commonly detected during 24–48 h Holter monitoring in HF patients with both ischemic and non-ischemic cardiomyopathies. In a small proportion of patients, non-sustained VT can produce symptoms, and, in such cases, treatment with antiarrhythmic drug therapy is appropriate. First-line antiarrhythmic therapy for symptomatic patients consists of optimization of β‑blockers; if this approach fails to control symptoms, amiodarone or sotalol are indicated [80]. The goal of acute therapy of sustained VT (defined by a duration of ≥30 s) is to rapidly restore a stable rhythm with a physiologically appropriate ventricular rate and thereby prevent organ damage or further hemodynamic deterioration. Patients with severe hypotension, chest pain, or evidence for hypoperfusion should be considered hemodynamically unstable, and direct current cardioversion is usually the most expeditious method for terminating the arrhythmia [81].

In many patients, however, the tachycardia is not immediately life-threatening and the patient is conscious and not in severe distress, in such patients pharmacologic cardioversion is the procedure of choice. Pharmacologic conversion of a sustained VT episode has not been studied in large controlled randomized trials; observational and limited controlled trial data, however, indicate that intravenous lidocaine (100–150 mg iv then slow iv infusion of a dose of 2–4 mg/min) has often been regarded as a first-line antiarrhythmic agent and can be useful in sustained VT associated with ischemia or myocardial infarction [82].

Intravenous procainamide (10 mg/kg iv) is an appropriate therapy in these patients, as it rapidly slows and terminates sustained VT; however, although procainamide is successful for acute arrhythmia termination in around 75% of patients with monomorphic sustained VT, its use can be limited by hypotension, which occurs in approximately 20% of these individuals [83].

Amiodarone (150–300 mg in 5 min iv, followed by an infusion of 1050 mg/day) is also useful, but its onset of action is slower than lidocaine or procainamide, and the results of acute termination studies have been variable [84].

Medical therapy is also indicated for secondary prevention of sustained VT/VF. Together with optimization of HF medical therapy amiodarone or sotalol must be used for reduce recurrence [85]. We recommend amiodarone (200 mg/day) as first line therapy and reserve the use of sotalol (240–480 mg/day in three times), together with beta-blockers, only in patients with contraindication/adverse effects to amiodarone or in patients with persistence of SVT despite amiodarone therapy. In case of recurrent sustained VT refractory to others antiarrhythmic drugs, mexiletine (600 mg/day in three times) can be used (not commercially available in some European countries such as Greece or Italy).

### 7.2. Catheter Ablation

Consensus document of European Heart Rhythm Association and Heart Rhythm Society’s on catheter ablation of VAs [86] lists the current indications for this procedure as symptomatic monomorphic sustained VT that recurs despite therapy with antiarrhythmic drugs or in patients who do not desire or are intolerant of antiarrhythmic medications. Catheter ablation is also indicated for patients with frequent PVCs, non-sustained VT or VT that results in myocardial dysfunction (due to tachycardia-related cardiomyopathy), and for patients with VT from bundle branch reentry or intrafascicular VTs and with recurrent sustained polymorphic VT or VF that is refractory to antiarrhythmic medications when there is a suspected trigger (single PVC morphology) that can be targeted for ablation.

#### 7.2.1. Ablation of Premature Ventricular Complex and Non-Sustained Ventricular Tachycardia

PVCs are common in patients with HF and structural heart disease. PVCs may also be accompanied by short runs of non-sustained VT. It is well documented that frequent PVCs may be the cause of a reversible cardiomyopathy. It is usually difficult to distinguish whether the PVCs are the primary problem or secondary to the cardiomyopathy. Cases in which PVCs represent more than 10% of heartbeats on a 24 h electrocardiogram, with most PVCs of the same QRS morphology, are more likely to induce tachycardiomyopathy [87]. PVC ablation in these patients may result in significant recovery of their left ventricular function.

#### 7.2.2. Ablation of Ischemic Cardiomyopathy

The mechanism of VT in patients with prior myocardial infarction is usually reentry around the myocardial scar, but focal mechanisms can occur in 5%–10% of patients. Early reperfusion strategies have resulted in smaller infarcts and less aneurysm formation but in patients with chronic ischemic cardiomyopathy, the risk of arrhythmia remains significant. Although programmed stimulation can induce VT in almost 90% of patients with ischemic cardiomyopathy and clinical sustained VT, the rate and morphology often differ from that of spontaneous occurring VT [88]. These factors have led to increasing utilization of substrate-based ablation, which uses voltage and pace mapping during sinus rhythm to identify targets for ablation. This minimizes the episodes of unstable VT during the procedure and is better tolerated by patients with reduced left ventricular systolic function. Most VT can be ablated from the endocardial approach, but some VT, more commonly prior to inferior infarction, may require an epicardial approach [89]. Outcomes of ablation vary considerably depending on the study population. Successful ablation is achieved in 38%–72%, with a procedural mortality of 0.5%–8% over 12 months; a follow-up of 50%–88% of patients will remain free of VT [90].

#### 7.2.3. Ablation of Non Ischemic Cardiomyopathy

Sustained monomorphic VT is less common in non-ischemic cardiomyopathy, but the mechanism is usually also reentry. Areas of scar in non-ischemic cardiomyopathy tend to be smaller and more often located in the mid-myocardium or epicardial layers. It is common to induce multiple morphologies of VT in this patient group and endocardial ablation alone is less successful [91]. Mapping and ablating epicardial sits increase the success in this patient group, but the optimal timing of epicardial ablation is yet to be determined [92].

## 8. Sudden Cardiac Death

### 8.1. Sudden Cardiac Death in Patients with HFrEF

Sudden, presumably arrhythmic death accounts for a significant proportion of total mortality in HF patients with II–III NYHA class, whereas progressive hemodynamic deterioration and pump failure are the major causes of death in patients with IV NYHA class. Many risk factors for arrhythmia recurrence and SCD have been identified in patients who have structural heart disease; however, developing a comprehensive risk stratification strategy remains a challenge. Here, we briefly illustrate the parameter used in clinical practice for stratification of SCD risk in HF patients.

#### 8.1.1. Left Ventricular Systolic Dysfunction

A reduced ejection fraction remains, according to international guidelines (Table 2), the most consistent predictor of SCD in patients who have structural heart disease, irrespective of etiology. Patients in the follow-up Multicenter Automatic Defibrillator Implantation Trial (MADIT-II) who had an ejection fraction less than 30% had a rate of SCD of approximately 9.4% at 20 months [93]. In a similar population of patients, however, an ejection fraction greater than 35% and history of myocardial infarction conferred only a 1.8% risk of SCD [94].

#### 8.1.2. T-Wave Alternans

T-wave alternans or beat-to-beat variation in the T-wave morphology is believed to be due to regional disturbances in action potential duration leading to dispersion in repolarization and propensity to develop arrhythmias [95]. Microvolt T-wave alternans (MTWA) measures microvolt changes in the T-wave amplitude in alternate beats and has also been found to be a significant predictor of VT/VF events [96]. Application of the MTWA test to patients who fit MADIT-II criteria demonstrated [97] that patients who had an abnormal MTWA test had a significantly increased 2-year mortality rate (17.8%) compared with patients who had a normal MTWA (3.8%); however, a major limitation of MTWA test is the high proportion of indeterminate results.

#### 8.1.3. Signal-Averaged Electrocardiogram

The signal-averaged electrocardiogram (SAECG) is a high-resolution electrocardiogram technique designed to determine the risk of developing VT by measuring the low-amplitude, high-frequency surface electrocardiogram signals in the terminal QRS complex that cannot be detected by a standard electrocardiogram machine [98]. These late potentials have been correlated to localized areas of delayed endocardial activation in humans and reflect the substrate for ventricular reentry [99]. In patients who have coronary artery disease, signal-averaged electrocardiogram has an overall low positive predictive value ranging from 7% to 27%, whereas it has a very high negative predictive value ranging from 96% to 99%. Its utility as a prognostic tool remains controversial in patients who have idiopathic non-ischemic cardiomyopathy [100].

In conclusion, we concur with the American Heart Association (AHA)/American College of Cardiology (ACC)/Heart Rhythm Society (HRS) scientific statement on non-invasive risk stratification [101], which concluded that the SAECG may be useful to identify patients at low risk for SCD, but its routine use to identify HF patients at high risk for SCD is not yet adequately supported.

#### 8.1.4. Study of Autonomic Tone

Heart rate variability and baroreflex sensitivity (BRS) are two non-invasive tests used to estimate the function of the autonomic nervous system. Decreased heart rate variability has been shown to be a powerful predictor of mortality and perhaps arrhythmic events in patients who have Myocardial Infarctions [102]. The Autonomic Tone and Reflexes after Myocardial Infarction trial was designed to evaluate the prognostic utility of BRS and heart rate variability in post-myocardial infarction patients [103]. A depressed BRS (defined as <3 ms/mmHg) significantly predicted cardiac mortality over an average 21-month follow-up period.

#### 8.1.5. Electrophysiologic Study

The diagnostic and prognostic values of an electrophysiology study depend on the underlying pathologic substrate and the spontaneous arrhythmia presentations. The inducibility of monomorphic VT is a powerful marker of risk for SCD, especially in patients who have a history of prior myocardial infarction and reduced ejection fraction or syncope. Programmed electrical stimulation has a sensitivity of about 97% in those who have spontaneous monomorphic sustained VT and a positive predictive value of 65% [104]. In patients who have non-ischemic cardiomyopathy, the inducibility of VAs is much lower. Although the overall sensitivity of programmed stimulation is similar to that in patients who have coronary artery disease, non-inducibility in patients who have non-ischemic dilated cardiomyopathies does not confer a good prognosis [105].

### 8.2. Sudden Cardiac Death in Patients with HFpEF

The incidence and mechanisms of SCD among patients with HFrEF have been well characterized, conversely limited data are available exploring the landscape of SCD in patients with HFpEF [106]. HFpEF is a heterogeneous clinical condition with an increasing prevalence and mortality and morbidity equal to that of HFrEF. Table 3 summarizes the epidemiological data, clinical predictors as well as strategies to prevent SCD in this population.

## 9. Management of Bradyarrhythmias in HF

**Figure 2:**
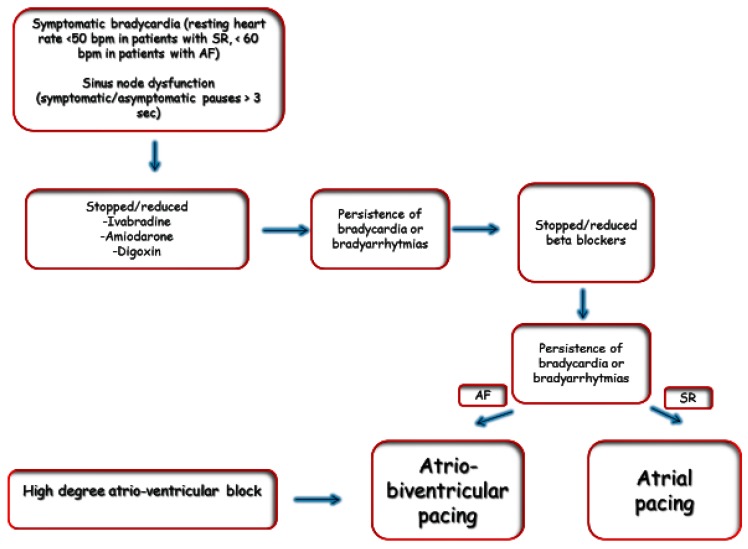
Flow chart for management of bradyarrhythmias in HF. AF: Atrial fibrillation; SR: Sinus Rhythm.

Bradyarrhythmias management in the HF population mirrored that in the general population, but careful individual decision-making is required in selecting the optimum pacing mode in patients with HF (Figure 2). In patients with sinus node dysfunction but intact atrio-ventricular conduction, atrial pacing alone (AAI mode) might be considered; on the other hand, patients with AV block (actual or threatened) will require ventricular pacing. There is no strong evidence from clinical trials that dual-chamber pacing (DDD or DDDR) is superior to single-chamber ventricular pacing (VVI or VVIR), even in the HF population. However, there is ample evidence that right ventricular pacing per se is deleterious in patients with left ventricular dysfunction [107]; for this reason, atrio-biventricular pacing is often considered in patients with HF and severe left ventricular dysfunction who are likely to require long-term ventricular pacing [108].

## 10. Conclusions

Clinical management of HF needs to consider the high risk of arrhythmias in these patients. Antiarrhythmic drug therapy is still suboptimal, but it is important for patients with symptomatic arrhythmias and atrial arrhythmias, and in selected patients with advanced HF. Implantable cardioverter defibrillator therapy effectively reduces mortality in patients with severely reduced ejection fraction; however, since only a minority of implanted patients will experience clinically relevant VAs, improved risk assessment for primary prophylaxis warrants further studies, including risk stratification in HFpEF.

## Figures and Tables

**Table 1:** Factors involved in pathogenesis of tachyarrhythmias in patient with heart failure.

***Structural and Hemodynamic Abnormalities***	Myocardial scar
	Left ventricular hypertrophy
	Left ventricular stretch
***Metabolic Abnormalities***	Neurohormonal activation
	Electrolyte abnormalities
***Electrophysiologic Changes***	Prolongation of action potential
	Changes of calcium homeostasis
	Changes of potassium current
***Others***	Pharmacologic agents
	Myocardial ischemia

**Table 2:** Indication to ICD implantation in HF patients. COR: Class of Recommendation.

***2012 ACC/AHA/HRS Guidelines for Device-Based Therapy of Cardiac Rhythm Abnormalities***	***COR-LOE***
Ischemic DCM (at least 40 days post-MI), LVEF less than or equal to 35%, NYHA functional Classes II or III	***I-A***
Non-ischemic DCM, LVEF less than or equal to 35%, NYHA functional Classes II or III	***I-B***
Ischemic DCM (at least 40 days post-MI) LVEF less than or equal to 35%, NYHA functional Class I	***I-A***
***2013 ACCF/AHA Guideline for the Management of Heart Failure***	
HFrEF (irrespective of etiology, but in case of ischemic etiology at least 40 days post-MI), LVEF less or equal to 35%, NYHA Classes II or III	***I-A***
HFrEF (irrespective of etiology, but in case of ischemic etiology at least 40 days post-MI), LVEF less or equal to 30%, NYHA Class I	***I-B***
***2015 ESC Guidelines for the management of patients with ventricular arrhythmias and the prevention of sudden cardiac death***	
Ischemic HFrEF (at least 6 weeks after myocardial infarction), LVEF less or equal to 35%, NYHA Classes II or III	***I-A***
Non ischemic HFrEF, LVEF less or equal to 35%, NYHA Classes II or III	***I-B***
Patients who are listed for heart transplant	***IIa-C***
***2016 ESC Guidelines for the diagnosis and treatment of acute and chronic heart failure***	
Ischemic HFrEF, LVEF less or equal to 35%, NYHA Classes II or III	***I-A***
Non-ischemic HFrEF, LVEF less or equal to 35%, NYHA Classes II or III	***I-B***

DCM: Dilated Cardiomyopathy; HFrEF: Heart Failure Reduced Ejection Fraction; LOE: Level of Evidence; LVEF: Left Ventricular Ejection Fraction; NYHA: New York Heart Association.

**Table 3:** Clinical aspect of sudden cardiac death in Heart Failure Preserve Ejection Fraction.

***Epidemiology of SCD in HFpEF***	39.4% of total cardiovascular death in CHARM-Preserved trial
	43.4% of total cardiovascular death in I-PRESERVE trial
	38.1% of total cardiovascular death in TOPCAT trial
***Factors associated to SCD risk in HFpEF***	Age *
	Male sex *
	History of diabetes mellitus *
	History of prior myocardial infarction *
	Left bundle branch block *
	Natriuretic peptides *
	Other Biomarkers (Galectin 3, soluble ST-2) **
***Strategy to prevent***	Clinical trials evaluating established therapies for patients with HFrEF in patients with HFpEF have not resulted in improvements in clinical outcomes. Trial with ARNI is ongoing (PARAGON-HF). Identification of specific phenotype (e.g., hypertrophic cardiomyopathy) is mandatory for tailored treatment

* Data derived from I-PRESERVE; ** Limited evidences are available; SCD: Sudden Cardiac Death; HFpEF: Heart Failure Preserved Ejection Fraction; ARNI: Angiotensin Receptor/Neprilysin Inhibitors; CHARM: Candesartan in Heart Failure Assessment of Reduction in Mortality and Morbidity; I-PRESERVE: Irbesartan in Heart Failure with Preserved Ejection Fraction Study; TOPCAT: Treatment of Preserved Cardiac Function Heart Failure with an Aldosterone Antagonist PARAGON-HF: Efficacy and Safety of LCZ696 Compared to Valsartan, on Morbidity and Mortality in Heart Failure Patients With Preserved Ejection Fraction.

## Abbreviations

The following abbreviation are used in the paper:

ACE-inhibitors	Angiotensin Converting Enzyme inhibitors
ARBs	Angiotensin Receptor Blockers
AF	Atrial Fibrillation
AFFIRM	Atrial Fibrillation Follow-up Investigation of Rhythm Management
AFL	Atrial Flutter
ARNI	Angiotensin Receptor/Neprilysin Inhibitors
AT	Atrial Tachycardia
AVRT	Atrio-Ventricular reentrant (or reciprocating) tachycardia
BRS	Baroreflex Sensitivity
CHARM	Candesartan in Heart Failure Assessment of Reduction in Mortality and Morbidity
COR	Class of Recommendation
DCM	Dilated Cardiomyopathy
ESC	European Society of Cardiology
HF	Heart Failure
HFrEF	Heart Failure Reduced Ejection Fraction
HFmEF	Heart Failure mid reduction Ejection Fraction
HFpEF	Heart Failure Preserved Ejection Fraction
ICD	Implantable Cardioverter Defibrillator
I-PRESERVE	Irbesartan in Heart Failure with Preserved Ejection Fraction Study
MADIT II	Multicenter Automatic Defibrillator Implantation Trial Investigator II
MRAs	Mineral Receptor Antagonists
MTWA	Microvolt T-wave alternans
NYHA	New York Heart Association
NSVT	Non Sustained Ventricular Tachycardia
PABA-CHF	Pulmonary Vein Antrum Isolation vs. AV Node Ablation With Biventricular Pacing for Treatment of Atrial Fibrillation in Patients with Congestive Heart Failure
PARAGON-HF	Efficacy and Safety of LCZ696 Compared to Valsartan, on Morbidity and Mortality in Heart Failure Patients with Preserved Ejection Fraction
PVCs	Premature Ventricular Complexes
RACE II	The Rate Control Efficacy in Permanent Atrial Fibrillation: a Comparison between Lenient versus Strict Rate Control II
SR	Sinus Rhythm
SVAs	Supraventricular arrhythmias
SVT	Sustained Ventricular Tachycardia
SCD	Sudden Cardiac Death
TOPCAT	Treatment of Preserved Cardiac Function Heart Failure with an Aldosterone Antagonist
Vas	Ventricular Arrhythmias
VT	Ventricular Tachycardia
VF	Ventricular Fibrillation
